# Barriers to the spiritual care of parents taking care of their child with a life-limiting condition at home

**DOI:** 10.1007/s00431-023-05314-4

**Published:** 2023-11-11

**Authors:** Marije A. Brouwer, Barbara C. Bas-Douw, Carlo J. W. Leget, Marijanne Engel, Saskia C. C. M. Teunissen, Marijke C. Kars

**Affiliations:** 1 Julius Center for Health Sciences and Primary Care, Center of Expertise in Palliative Care Utrecht, University Medical Center Utrecht, Universiteitsweg 100, 3584 CG Utrecht, The Netherlands; 2 Department of Care Ethics, University of Humanistic Studies, Utrecht, The Netherlands

**Keywords:** Grief, Palliative care, Child care, Parents, Spirituality, Focus groups

## Abstract

The changes that parents face when caring for a child with a life-limiting condition at home can affect them on a spiritual level. Yet, indications remain that parents do not feel supported when dealing with spiritual issues related to caring for a severely ill child. This paper explores, from the perspectives of bereaved parents, chaplains, grief counselors, and primary health care providers, the barriers to supporting the spiritual needs of parents. We conducted a qualitative focus group study from a constructivist point with chaplains/grief counselors, primary care professionals, and bereaved parents. All groups participated in two consecutive focus group sessions. Data were thematically analyzed. Six chaplains/grief counselors, 6 care professionals, and 5 parents participated. We identified six barriers: (1) There were difficulties in identifying and communicating spiritual care needs. (2) The action-oriented approach to health care hinders the identification of spiritual care needs. (3) There is an existing prejudice that spiritual care needs are by nature confrontational or difficult to address. (4) Spiritual support is not structurally embedded in palliative care. (5) There is a lack of knowledge and misconceptions about existing support. (6) Seeking out spiritual support is seen as too demanding.

*Conclusion*: Parents of children with life-limiting conditions face existential challenges. However, care needs are often not identified, and existing support is not recognized as such. The main challenge is to provide care professionals and parents with the tools and terminology that suit existing care needs.

**Table:** 

**What is Known:** *• Spiritual care needs are an important aspect of pediatric palliative care.* *• Parents of children with life-limiting conditions feel unsupported when dealing with spiritual questions.*	
**What is New:** *• Parents and professionals mention barriers that hinder spiritual support for parents.* *• There is a disconnect between existing support and the care needs that parents have.*

**What is Known:**

*• Spiritual care needs are an important aspect of pediatric palliative care.*

*• Parents of children with life-limiting conditions feel unsupported when dealing with spiritual questions.*

**What is New:**

*• Parents and professionals mention barriers that hinder spiritual support for parents.*

*• There is a disconnect between existing support and the care needs that parents have.*

## Introduction

Parents’ lives are affected on many levels by the reality that their child suffers from a life-limiting condition. The hopes and dreams they might have for their child and themselves alter radically [1–4]. Parents are often deeply involved in their child’s medical care, which, in many cases, has an impact upon their professional careers and social lives[4–6]. In addition to this, parents may have to deal with the prospect of losing their child [1, 7, 8]. These changes can affect parents deeply on a spiritual level.

Addressing spiritual needs is part of pediatric palliative care, as spirituality is one of its four dimensions [9, 10]. In the Netherlands, chaplains^1^ and grief and bereavement counselors are available to offer specialist spiritual support to parents. There have been rapid developments in pediatric palliative care, the availability of professional support, and the inclusion of the spiritual domain in national guidelines [11, 12], partly based on the guideline “End of life care for infants, children and young people” of the British National Institute for Health and Care Excellence (NICE) [13]. Yet despite this, the scarce research in this area shows that both in the Netherlands and other countries, spiritual support in pediatric palliative care, including the involvement of chaplains or grief and bereavement counselors, is still not self-evident [14, 15]. Indications remain that parents do not feel properly supported when dealing with the spiritual issues of parenting and caring for a severely ill child [3, 16, 17].

### Theoretical background

Spirituality is defined by the EAPC Reference Group on Spiritual Care as “the dynamic dimension of human life that relates to the way persons (individual and community) experience, express and/or seek meaning, purpose and transcendence, and the way they connect to the moment, to self, to others, to nature, to the significant and/or the sacred” [18].

### Aim

The aim of this study was to explore, from the perspectives of bereaved parents, chaplains, grief counselors, and primary health care providers, the barriers to supporting the spiritual needs of parents.

## Methods

### Design

From a constructivist point, we conducted a focus group study with three homogeneous groups, consisting of bereaved parents, chaplains/grief counselors, and primary care professionals, in order to identify barriers to spiritual support from a diverse perspective [19]. Data were analyzed thematically. The study was reviewed and judged to be exempt from the Medical Research Involving Humans Act by the medical ethics committee of the University Medical Center Utrecht (number 20/533, August 2020). We followed the COREQ Consolidated criteria for reporting qualitative research [20].

### Sample

Chaplains, grief counselors, and primary care professionals, currently involved in pediatric palliative care, were eligible for participation in this study if they were practicing their profession in the Netherlands and had recent (< 1 year) experience supporting parents of a child with a life-threatening condition. Maximum variation was sought with regard to experience and affiliation. Parents (including biological parents, adoptive parents, and foster parents) were eligible when they had cared for their child at home and the child (0–21) had died less than 3 years prior to the focus group. Maximum variation was sought with respect to diagnosis, gender, and age of the child.

Chaplains, grief counselors, and primary care professionals were recruited via existing professional networks. Parents were recruited via open recruitment on online platforms of parent support groups by spreading a call for participants describing the aim of the study, what parents we looked for, and researcher contact details. Participants were not compensated for their participation in the focus groups.

If interested chaplains, grief counselors or parents contacted the researchers, a telephone intake took place to check whether participants met the criteria to participate, and written informed consent was given by all participants prior to the focus groups.

### Data collection

Two rounds of three focus group meetings were held. All groups participated in two consecutive homogenous focus group sessions. In the first round, participants shared barriers that they perceived from their perspective as professionals or parents. A thematic analysis was performed between both rounds to summarize the main outcomes. In the second round, the participants were given insights into the outcomes of the different disciplines or parties that participated in the other focus group sessions. Here, they were asked to reflect upon barriers mentioned by the other groups. This design was chosen to obtain a variety of perceptions in a safe environment [21], while also allowing participants to explore and debate the perspectives of other parties. All focus groups were held in Dutch. Translation of quotes and analysis were performed by the research team and checked by a language editing service.

Focus groups were held online on Cisco Webex due to COVID-19. The focus group meetings were structured using a written topic guide developed according to the structure provided by Krueger and Casey [19, 21]. Meetings were guided by two interviewers and an observer, all three female. These roles circulated between BBD, MAB, and MCK. The meetings were audio and video recorded and transcribed verbatim. The participants received a summary of the output of the other groups between the first and the second meetings and were asked to reflect on the findings of the other groups in the second meeting.

### Data analysis

Data from both focus group rounds were analyzed using an inductive, thematic approach [22]. Three researchers (BBD, MAB, and MCK) read all transcripts and field notes in order to become familiar with the data. Meaningful fragments were identified through open coding. Subsequently, codes were identified through latent coding. The text was coded (BBD) using the software program NVivo. Two researchers (MAB, MCK) checked the coding and analysis. Coding and interpretations from both focus group rounds were compared and after the second round categories found in the first round were nuanced or sharpened where necessary. These resulted in broader themes. Researcher triangulation was ensured throughout the process by involving multiple researchers with different backgrounds in all stages of the study design, data collection, and data analysis, in order to improve the reliability and validity of the analysis and limit bias. Any differences were settled by a discussion within the research group until a consensus was achieved.

## Results

### Participants

The first focus group consisted of three chaplains and three grief counselors. The second group consisted of one general practitioner, two employees of a medical day care center, and two pediatric primary care nurses. The third focus group was composed of one father and four mothers. Due to scheduling difficulties, one chaplain, one grief counselor, and one parent missed out on the second focus group meeting. For characteristics, see Table 1.

**Table 1:** Participant characteristics

Characteristics of chaplains and grief counselors (*N* = 6)	
**Affiliation**	
Hospital	2
Own practice	4
**Religious background**	
None	3
Christian	2
Islam	1
**Years of experience**	
< 5 years	1
5–15 years	2
> 15 years	3

Characteristics of health care professionals (*N* = 6)	
**Profession**	
General practitioner	1
Pediatric primary care nurses	3
Employee medical daycare center	2
**Years of experience**	
< 5 years	1
5–15 years	2
> 15 years	3

Characteristics of parents (*N* = 5)	
**Parent**	
Mother	4
Father	1
**Time past since bereavement at moment of FG**	
< 1 year	1
1–2 years	1
2–3 years	3
**Diagnosis of child**	
Malignancy	2
Non-malignancy	3

From the data from both focus group rounds, we identified six major barriers to providing spiritual support for parents in pediatric palliative care. (1) There were difficulties in identifying and communicating spiritual care needs. (2) The action-oriented approach to health care hinders the identification of spiritual care needs. (3) There is an existing prejudice that spiritual care needs are by nature confrontational or difficult to address. (4) Spiritual support is not structurally embedded in palliative care. (5) There is a lack of knowledge and misconceptions about existing support. (6) The final barrier was that seeking out spiritual support is seen as too demanding. Quotes for all themes are presented in Table 2.

**Table 2:** Quotes

**Barrier**	**Quote**
1. Difficulties in identifying and communicating spiritual care needs.	**Grief counselor, round 1.** “It is not easy to unveil spirituality. I think that’s one of the barriers of the spiritual dimension, that it’s hidden in stories people tell about their experiences. That’s why it is difficult to identify, because parents themselves do not always recognize it as such.”
	**Primary care professional, round 1.** “The signals that parents give off are often very small and hardly noticeable, and you have to respond adequately at these moments, and sometimes, these are the only moments where that is possible. But that moment might pass quickly, and then you’ve lost your opportunity for a conversation.”
	**Parent, round 2.** “Spirituality… I also had to think about that word. What does that mean to me? But if you talk about what makes you happy? What brings you joy? What gives you energy? those are questions that you can answer much easier.”
2. The action-oriented approach to health care hinders the identification of spiritual care needs.	**Primary care professional, round 2.** “I think that the issue of nurses is that when we see a problem, we immediately want to solve it. That is more or less the core of our profession. But through the years I’ve learned that I sometimes have to sit back and listen.”
	**Parent, round 1.** “At medical day care, goals had to be achieved. Whereas, we thought: “Well, we are already happy if he just has a comfortable day.” (…) So it is so important the comfort of the child is being taken into account. And, I actually think that goes hand-in-hand, for me at least, with meaningfulness.”
	**Grief counselor, round 2.** “I think that in the medical setting, the immeasurability affects [the lack of focus on spirituality]. But life itself is not measurable. The meaning of life if not measurable, you can’t put it into tables and charts. The only thing you can do is sit down with someone and take your time.”
3. There is an existing prejudice that spiritual care needs are by nature confrontational or difficult to address.	**Parent, round 1**. “In the beginning [the conversations] were very confrontational for us, because […] we just were not ready for it yet. And suddenly, we had conversations about whether or not to resuscitate and things like that. So, initially we were very hesitant, and dug our heels in a little.”
	**Grief counselor, round 2.** “It is very dependent on how familiar the physician is with the end of life. A physician with experience, who is familiar with it, or comfortable, may realize that it doesn’t have to be an emotional burden to talk about it, and that death can have beautiful aspects as well.”
	**Chaplain, round 2.** “I think that’s where the bottom line is, because they don’t align properly with what’s significant for those parents, they sometimes bring up treatment restrictions when parents aren’t ready, have no idea, or too soon, or too late.”
4. Spiritual care needs are not structurally embedded in palliative care.	**Primary care professional, round 1**. “I have noticed that it is very dependent on the individual professional. Parents can meet someone who sees it as an important topic and who is willing to talk about it from an early stage. But, I also notice that parents very often meet doctors who find it difficult to talk about it, and therefore address it way too late.”
	**Parent, round 2.** “The difficulty is that professionals don’t know what has been talked about with whom. Because we’re missing a single system, where all involved can read back the documentation of conversations. (…) So if you have certain conversations, other professionals won’t know about it. That’s problematic.”
	**Chaplain, round 2.** “For me, here lies the heart of the problem, that we’re not part of the core team. And I also don’t know if we could fix that easily.”
5. A lack of knowledge and misconceptions about existing support.	**Parent, round 1.** “I came into contact with a chaplain, and initially I was very hesitant. I thought, I wouldn’t do spiritual counseling, and that, I didn’t need that at all. But once we had made an appointment, (…) I could discuss everything with her. And she also gave me the confidence that I could discuss all those difficult things that were going on in my head with her.”
	**Chaplain, round 1.** “There are many families who don’t know about spiritual care. [And when I talk with them about spirituality], they will look at me and say “no, I am not religious.” And then you have to explain that it doesn’t necessarily have to be related to religion.”
	**Primary care professional, round 2.** “For me, I’ve noticed that parents flinch when hearing the term ‘grief counselor’. And colleagues too, because they associate it with sadness and death. But the loss of the health of your child because your child has an illness, or the loss of your own freedom, they don’t really associate the term with that. So I think that we should provide more information there."
6. Seeking out spiritual support is seen as too demanding.	**Chaplain, round 1.** “I asked parents if they had spoken to chaplains. They all said no. And then I asked why not? Weren't there spiritual or existential questions? And I learned that they understood spiritual questions, as questions about ‘why [things happened]’…. and that is a question that you can’t handle as a parent […] it undermines your strength. […] If you were to ask parents in that situation instead: ‘What is important to you? What is of value, what is meaningful to you?’ then it would have been beneficial.”
	**Primary care professional, round 2.** “I see that for a lot of parents, there is a threshold that they need to overcome to go to a grief counselor or chaplain. And that threshold it quite high. And we can help them by providing information and to provide them with a network of support, but still, the threshold remains high.”
	**Parent, round 2.** “What prevented me [from getting support], is that you always have to go somewhere. So you have to go to a hospital or you have to go to a practice…. But parents generally want to focus on other things; taking care of their child, but also doing nice things, making memories.”

### Difficulties in identify and communicate spiritual care needs

A significant barrier mentioned by all participants was a lack of a unified language with regard to spiritual care needs. This hindered both parents and professionals in identifying and communicating these care needs. Respondents had difficulties in defining what spiritual care needs were and reported finding it difficult to identify spiritual care needs. While parents experienced many questions relating to the spiritual dimension, these were not identified as such. They involved questions such as the following: “What is important for my child? What gets me out of bed? What makes my days worthwhile? What do I want to pursue in life?” Because professionals and parents themselves did not identify and express these experiences as care needs, support was usually not explicitly sought or offered.

Furthermore, participants stated that existing terminology was unsuitable. Parents shared the view that terms like “spirituality,” “giving meaning,” and “existential questions” were confusing and implied additional transcendental or religious meanings which they could not relate to their own experiences.

### The action-oriented approach to health care hinders the identification of spiritual care needs

Participants shared that the instinct of health care professionals is to provide practical solutions and to help the parent. They perceived it to be beneficial for medical care but felt it might complicate attempts to identify and address spiritual care needs. This sometimes hindered their ability to take a step back, to listen, and to offer parents a space to evaluate and reflect on their own thoughts and needs. Chaplains and grief counselors related that spiritual care needs were seldom expressed in a straightforward manner. It took time, listening skills, and sensitivity to identify these types of care needs.

A chaplain concluded that, in general, there was insufficient awareness in health care that a child’s life-limiting condition triggers spiritual questions. The focus in health care was on treating the patient rather than on the spiritual dimension of the well-being of patients and families. A distinct element of the approach of health care professionals, orientated towards action, was a focus on achieving treatment goals. Parents felt that too little attention was given to what was valuable and meaningful for them and their child, and whether the care given aligned to these values.

### There is an existing prejudice that spiritual care needs are by nature confrontational or difficult to address

When talking about spiritual care needs, health care professionals and parents, initially, had a tendency to focus on complex issues, such as end-of-life decision-making, ethical dilemmas, or the quality of life. From this perspective, they deemed that addressing spiritual topics might be difficult, delicate, and confronting.

Regularly, emotions, shame, or fear of confrontation hampered communication, while cultural differences might add to making such topics difficult to talk about. It also hinders health care professionals from fully embracing these conversations as part of their professional responsibilities. Chaplains and grief counselors emphasized that while these topics might be difficult, talking about what truly mattered to parents did not have to be connected to difficult topics. For example, conversations on treatment goals and end-of-life decisions implied an underlying layer of the values that parents have. These might include what they think is good and valuable for their child and what are their wishes, goals, or fears. The conversation about such a topic might empower parents instead of confronting them with conversations they are not yet ready for.

### Spiritual support is not structurally embedded in palliative care

While all the participants acknowledged the importance of giving attention to the spiritual care needs of parents, they observed a lack of regular conversations aimed at achieving this. Families dealt with a wide variety of caregivers and organizations and so it was unclear who was responsible for monitoring and addressing spiritual needs. As they were not an inherent part of the care given, whether or not conversations took place, and what was their quality, depended mainly on the individual caregiver or the efforts of parents to seek out support for themselves.

Participants suggested allocating one caregiver to address spiritual care needs informally, but on a regular basis, and to refer parents to a chaplain or grief counselor if necessary.

### There is a lack of knowledge and misconceptions about existing spiritual support

All participants thought spiritual and grief counseling could be valuable forms of support for parents. But, parents and health care professionals often lacked a clear idea of how this support from chaplains and grief counselors could be beneficial for parents. Furthermore, care professionals were hesitant to refer parents, because they were not familiar with the grief counselors or chaplains in their area and thus could not assess their contribution nor the quality of their support.

The terms used to identify the professionals in spiritual care were a major reason why parents and health care professionals were unfamiliar with the expertise of chaplains and grief counselors as sources of support for spiritual care needs.

Most parents and primary care workers reported the term “chaplains” implied religion, suggesting that their expertise lay solely in guiding parents with religious questions.

The term grief counselors suggested a focus on dying and supporting bereaved parents, which evoked negative connotations. A primary care professional added that by suggesting a parent should seek help from a grief counselor might upset parents, or give them the wrong message about their child’s prognosis.

Some chaplains and grief counselors acknowledged that they avoided using their job titles because of this. They stressed the importance of clear and appealing language to describe the contribution spiritual and grief counseling can make to parents with spiritual care needs, regardless of their religious preference, or the stage of their child’s illness.

### Seeking out spiritual support is seen as too demanding

Parents in our focus group acknowledged the importance of addressing spiritual care needs at an early stage. However, they also stressed that during the disease, spiritual care needs might often be met better by practical support than by traditional therapeutic sessions. They perceived therapeutic sessions as demanding too much time and energy, energy that parents could also have spent with their children, or on activities that brought joy and meaning to their lives.

A chaplain acknowledged this point and emphasized that during the course of the illness, one should not conduct demanding therapeutic sessions, but rather help parents in seeking, as far as possible, meaning in their daily lives.

Parents suggested that conversations on spiritual topics should be as informal as possible in order for parents not to feel overburdened by yet another demand in their lives. These conversations could take place in their home environment or during shared activities, such as during a walk. They considered sensitivity on the part of the professional, and connection on a human level, more important than a specific job title.

## Discussion

This focus group study into barriers to support parents with spiritual care needs shows that the reason why support is lacking is not because parents don’t have care needs, nor because support is not available. But, rather, it is because both care needs and support are not being recognized.

Dealing with their child’s illness and future prospects creates major challenges for parents with regard to who they are, what is important for them, and how they deal with their child’s illness and future prospects. Earlier studies give insight into particular challenges that parents face. Staying hopeful can be challenging [23, 24]. Parents often feel lonely and experience a general lack of understanding and sensitivity [25, 26]. They struggle to find new ways to express themselves as parents [27–29].

Parents and health care professionals do not identify the spiritual dimension of the care needs of the parents, and, thus, they do not seek support. Leaving these spiritual challenges unaddressed may leave parents feeling lonely and overwhelmed. At the same time, it may create a feeling of helplessness in health care professionals who see parents struggle but do not know how to support them [15]. Chaplains and grief counselors feel that they can offer such parents support, but feel that, especially in home settings, their expertise is unknown.

Why might it be the case that while both demand and support exist, the connection between the two seems to get lost? A reason for this may lay in the way that both palliative care and society have changed over the last decades.

Developments in pediatric palliative care are relatively recent [30, 31]. Amid these developments, existing professionals have to relate to this field and the specific demands that come with it. While studies show that the spiritual dimension, and how parents give meaning to their child’s illness, is an important aspect of caring for a child with a life-limiting condition [32], spiritual care has been largely underrepresented in the developments in pediatric palliative care. The question is how these elements can become an inherent part of pediatric palliative care.

Spirituality in palliative care has historically been closely connected to religion, and chaplains have been a major source of spiritual support since before the rise of palliative care [33]. However, the relation between spirituality and religion has changed. Over the last decades, the religious landscape in many Western countries, including the Netherlands, has become more secular, while immigration has brought more religious diversity. This transition is reflected in de spiritual care needs of families in pediatric palliative care. As is shown in this study, religion is only part of the spiritual dimension for parents, and spiritual care needs encompass a wide range of aspects of human experience, both of a religious and non-religious nature. This broad approach to spirituality is also reflected in the EAPC definition of spirituality [18]. Many chaplains and other spiritual workers are equipped to deal with the spiritual care needs of both a religious and non-religious nature. But while societal demands, and the content of their profession, have changed radically, the association that their job title evokes, unfortunately, has not, preventing parents from identifying support.

Other factors, such as a lack of funding to provide spiritual care in home settings until 2019 [34], have also hindered support. In the relatively new field of pediatric palliative care, chaplains and, similarly, grief counselors face the task of making sure that the way they position themselves as a source of support that is recognizable and accessible matches today’s needs and expectations.

### Strengths and limitations

One limitation was that the focus groups needed to be held in a digital setting due to COVID-19. The digital setting did however enhance possibilities for participants to participate [35], whereas the size of the groups was limited to ensure a good conversation [36]. Second, although we succeeded in including chaplains and grief counselors from various religious and cultural backgrounds, we did not succeed in including parents with such backgrounds, defined as non-Western, in the focus group. The strength of the study lies in the multi-dimensional design, which enabled us to gather the perspectives of parents, primary care workers, chaplains, and grief counselors. Secondly, because we held two rounds of focus groups, we were able to let participants share their own experiences, as well as reflect on barriers mentioned by the other groups. This enabled us to conduct a much more in-depth synthesis of existing barriers to spiritual support.

## Conclusion

Parents of children with life-limiting conditions face serious existential challenges which may result in them needing spiritual support. But, due to the fact that language surrounding this topic is obscure, and that spiritual support is not an inherent part of the care structure, care needs are often not identified, and existing support is not recognized as such. We want to recognize, acknowledge, and address the spiritual care needs of parents and offer suitable support where it is needed. Therefore, the main challenge is not to add new support systems, but to bridge the gap between the spiritual care that already exists, and the spiritual care needs of parents that often go unnoticed.

## Data Availability

The data of this study are kept by M.C.K. in the University Medical Center Utrecht, Utrecht, the Netherlands, and are available upon reasonable request.

## Footnotes

1 In this article, we use the term “chaplain” to refer to the group of professionals known in Dutch as “geestelijk verzorgers” (literal translation: spiritual carers). While similar, these professions are not entirely the same, as the care provided by geestelijk verzorgers covers a broader range of care needs connected to spirituality and is not necessarily religious by nature.
